# Effect of the size of halide ligands on the crystal structures of halide-bibridged polymers of Hg*X*_2_ with 4-ethyl­pyridine

**DOI:** 10.1107/S2053229625009702

**Published:** 2025-11-10

**Authors:** B. M. Parveen Beebeejaun-Boodoo

**Affiliations:** a Department of Chemistry, University of Pretoria, Private Bag X20, Hatfield, 0028, Pretoria, South Africa; Rigaku Americas Corporation, USA

**Keywords:** coordination polymers, crystal structure, halide-bibridged polymers, mercury, ethylpyridine, organic-inorganic hybrid

## Abstract

Halide-bridged polymers are a type of coordination polymer whereby halide ligands act as bridging ligands between the metal centres. The crystal structures of three halide-bibridged polymers of the formula [Hg(μ-*X*)_2_(4-Etpy)]_*n*_, namely, *catena*-poly[[(4-ethyl­pyridine)­mercury(II)]-di-μ-halido], ob­tained through the combination of the organic ligand 4-ethyl­pyridine (4-Etpy, C_7_H_9_N) and Hg*X*_2_ (*X* = Cl, Br or I), were determined. In these structures, abbreviated as **4epHgCl**, **4epHgBr** and **4epHgI**, respectively, the Hg^II^ ion ex­hibits a coordination number of five. All three structures were found to display a similar one-dimensional scalloped polymeric chain with halide ligands bridging pairs of Hg^II^ ions in a bidentate fashion; however, **4epHgI** differs from the other two structures in terms of the packing arrangement of the polymer. The change of the halide ligand to the larger iodide ligand disrupts the formation of the regular halide-bibridged polymeric chain observed in the chloride and bromide analogues, with **4epHgI** displaying pseudo-bridging in the polymer chain.

## Abstract 2

Three pyridine-based halide-bibridged polymers have been synthesized and their structures determined. The effect of the size of the halides on the crystal structures has been investigated.

## Introduction

Halide-bridged polymers are a subgroup of coordination com­pounds com­prising metal ions that are linked *via* bridging halide ligands into a polymeric structure. The metal ions are typically also coordinated to additional organic ligands, resulting in a polymer of the formula [*M*(μ-*X*)_*y*_(*L*)_*z*_]_*n*_, with *M* indicating the metal ion, *X* the bridging halide ligand and *L* the organic ligand. Additional terminal halide ligands may also be present in these polymers. The structural diversity dis­played by halide-bridged polymers make them excellent candidates for use in crystal engineering studies.

Halide-bridged polymers display physical properties, such as magnetic exchange and electrical conductivity along the halide-bridged polymer, as well as luminescence, catalytic activity and non-linear optical properties (Givaja *et al.*, 2012 ▸; Eckberg *et al.*, 1975 ▸; Crawford *et al.*, 1977 ▸; Estes *et al.*, 1978 ▸; Zhang *et al.*, 1997 ▸; Wei *et al.*, 1996 ▸; Slabbert *et al.*, 2015*a* ▸). Of specific inter­est in the current study are halide-bridged polymers containing the metal ion Hg^II^ and the organic ligand 4-ethyl­pyridine (4-Etpy).

The Hg^II^ ion shows a range of coordination configurations ranging from trigonal planar, square-planar and octa­hedral to the preferred tetra­hedral environment due to its softness and fully filled orbitals, allowing it to bind with soft anions such as Cl^−^, Br^−^ and I^−^ (Slabbert *et al.*, 2015*b* ▸; Englert *et al.*, 2010 ▸; Hu *et al.*, 2007 ▸). The coordination around the Hg^II^ cations allows for a wide range of bonding distances to potential donor atoms (Englert *et al.*, 2010 ▸). Halide-bridged polymers of divalent *d*^10^ metal cations with N-donor ligands have been studied, focusing on the effect of electronically modified pyridine/pyrazine-derived N-donor ligands on the structure of the coordinated halide-bridged chain (Hu *et al.*, 2001 ▸, 2003 ▸; Wang *et al.*, 2009 ▸; Mahmoudi *et al.*, 2009 ▸; Morsali *et al.*, 2009 ▸). The size of the N-donor organic ligand has an effect on the halide-bridged polymer and the extent to which the width of the coordinating N-donor organic ligand can be increased without disrupting the chain of the halide-bridged polymer has also been investigated (Slabbert *et al.*, 2015*b* ▸).
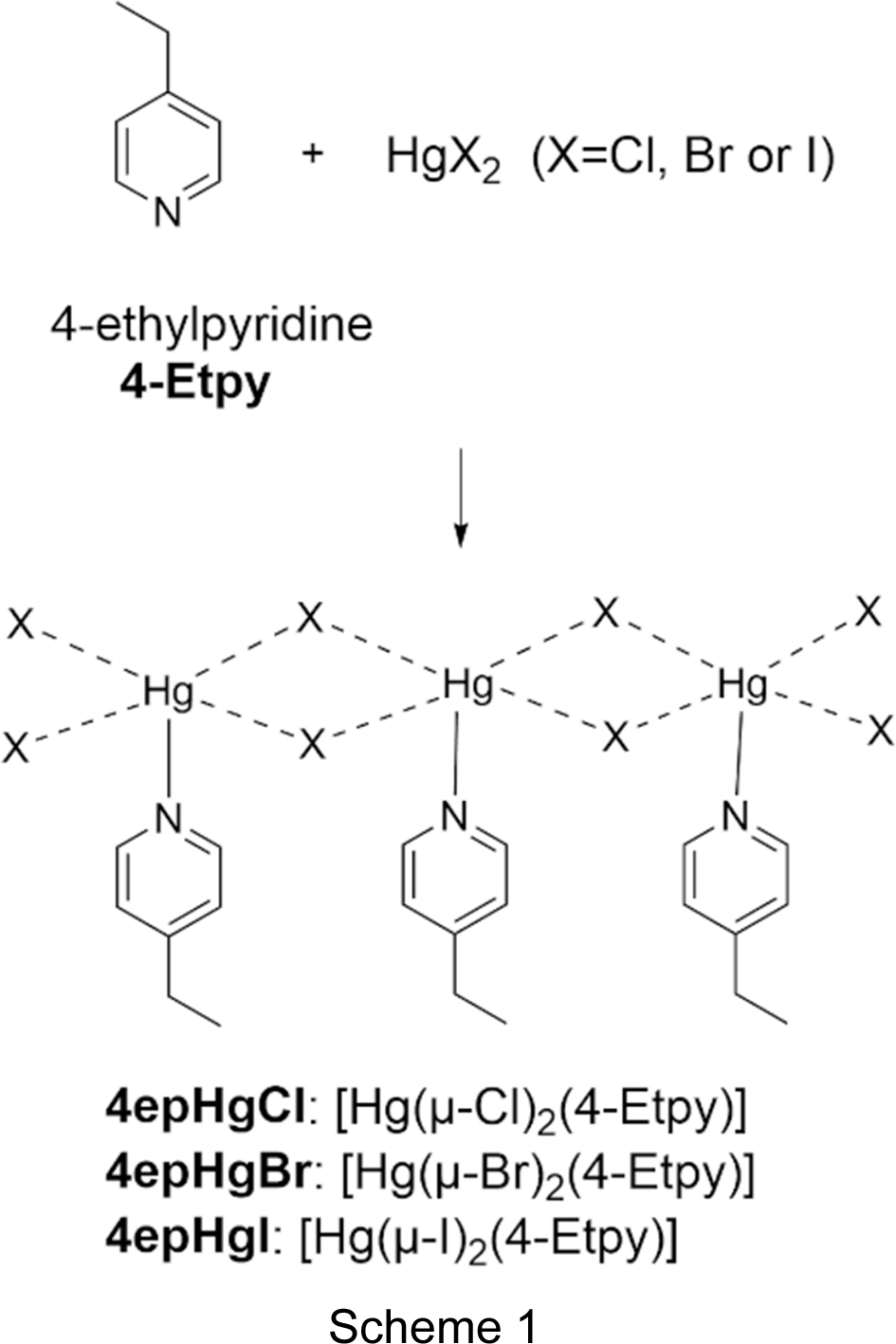


A one-dimensional polymeric structure with com­position [Hg(μ-Cl)_2_(pyridine)_2_]_*n*_ was reported for the combination of pyridine with HgCl_2_, and the structure obtained is a chloride-bridged polymeric lattice of HgCl_4_ square-planar entities with pyridine ligands occupying the *trans*-octa­hedral sites around the Hg^II^ ion (Canty *et al.*, 1982 ▸). However, when the halide was changed from Cl^−^ to Br^−^ and I^−^, no polymer was formed. The reactions of Hg*X*_2_ (*X* = Cl, Br or I) with the organic ligand 2-(2-hy­droxy­eth­yl)pyridine also formed one-dimensional poly­meric chains of [(*X*)Hg(μ-*X*)_2_{2-(2-hy­droxy­eth­yl)pyridine}]_*n*_ (Mobin *et al.*, 2010 ▸). In these polymers, the organic ligand binds to the Hg^II^ ion in a monotopic fashion *via* only the pyridine N-atom donor, leaving the –(CH_2_)_2_OH group at the *ortho* position as a pendant ligand. The halide-bridged poly­mer can either adopt a planar or a zigzag motif due to the flexibility of the Hg^II^ metal centre. If the bridging halide ligands of adjacent Hg^II^ ions are not arranged in a coplanar fashion, then a zigzag structure is favoured (Englert *et al.*, 2010 ▸).

A search of the Cambridge Structural Database (CSD, Version 5.46, February 2025 update; Groom *et al.*, 2016 ▸) revealed that no crystal structure containing the organic ligand 4-ethyl­pyridine and Hg^II^ has been reported in the literature. This indicated a gap in the literature, prompting the current investigation. This present study focuses on the organic–in­or­ganic hybrid com­pounds formed by the reaction of Hg*X*_2_ (*X* = Cl, Br or I) with the N-donor ligand 4-ethyl­pyridine (4-Etpy), as illustrated in Scheme 1. The structures of the com­pounds formed are abbreviated as **4epHg*****X***, with **4ep** re­pre­senting the organic ligand and **Hg*****X*** referring to the halide-bridged portion of the polymer. These reactions resulted in the formation of three new one-dimensional halide-bibridged polymers, namely, *catena*-poly[[(4-ethyl­pyridine)­mercury(II)]-di-μ-chlorido], **4epHgCl**, *catena*-poly[[(4-ethyl­pyridine)­mer­cury(II)]-di-μ-bromido], **4epHgBr**, and *catena*-poly[[(4-ethyl­pyridine)­mercury(II)]-di-μ-iodido], **4epHgI**.

## Experimental

### Chemicals and reagents

All chemicals were used as purchased without further puri­fi­cation: HgCl_2_ (98%, Fluka), HgBr_2_ (98%, Sigma–Aldrich), HgI_2_ (99%, Riedel de Haen), 4-ethyl­pyridine (98%, Sigma–Aldrich), ethanol (EtOH) (99.5%, Merck), methanol (MeOH) (99%, Merck) and tetra­hydro­furan (THF) (99.9%, Sigma–Aldrich).

### Synthesis and crystallization

#### Synthesis of 4epHgCl

A solution of HgCl_2_ (0.954 mmol, 0.2589 g) dissolved in EtOH (5 ml) was added to a slightly heated and stirred solution of 4-ethyl­pyridine (0.961 mmol, 0.1030 g) dissolved in EtOH (10 ml). The resulting solution was heated for 15 min and left at room tem­per­a­ture, open to the atmosphere, to crystallize. A batch of colourless rod-like crystals of **4epHgCl** were harvested (yield 57%) upon formation after two weeks.

#### Synthesis of 4epHgBr

A solution of HgBr_2_ (0.933 mmol, 0.3363 g) dissolved in THF (7 ml) was added to a slightly heated and stirred solution of 4-ethyl­pyridine (0.944 mmol, 0.1012 g) dissolved in MeOH (10 ml). The resulting solution was heated for 15 min, covered with Parafilm and left to crystallize at room tem­per­a­ture. A batch of colourless rod-like crystals of **4epHgBr** were har­vested (yield 62%) upon formation after two weeks.

#### Synthesis of 4epHgI

A solution of HgI_2_ (0.940 mmol, 0.4270 g) dissolved in THF (7 ml) was added to a slightly heated and stirred solution of 4-ethyl­pyridine (0.950 mmol, 0.1018 g) dissolved in MeOH (10 ml). The resulting solution was heated for 15 min, covered with Parafilm and left to crystallize at room tem­per­a­ture. A batch of colourless needle-like crystals of **4epHgI** were har­vested (yield 59%) upon formation after two weeks.

### Refinement

Crystal data, data collection and structure refinement details are summarized in Table 1 ▸. The structures of **4epHgCl** and **4epHgI** were refined as two-com­ponent inversion twins with the twin law (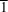
00 0
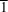
0 00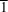) applied to both. The BASF was refined to 0.186 for **4epHgCl** and 0.026 for **4epHgI**. The inclusion of the twin refinement improved the quality of the models, as evidenced by a decrease in the *R* factor. The riding model was employed to position the H atoms in the three structures.

## Results and discussion

### Crystallographic discussion of the structures

**Figure 1:**
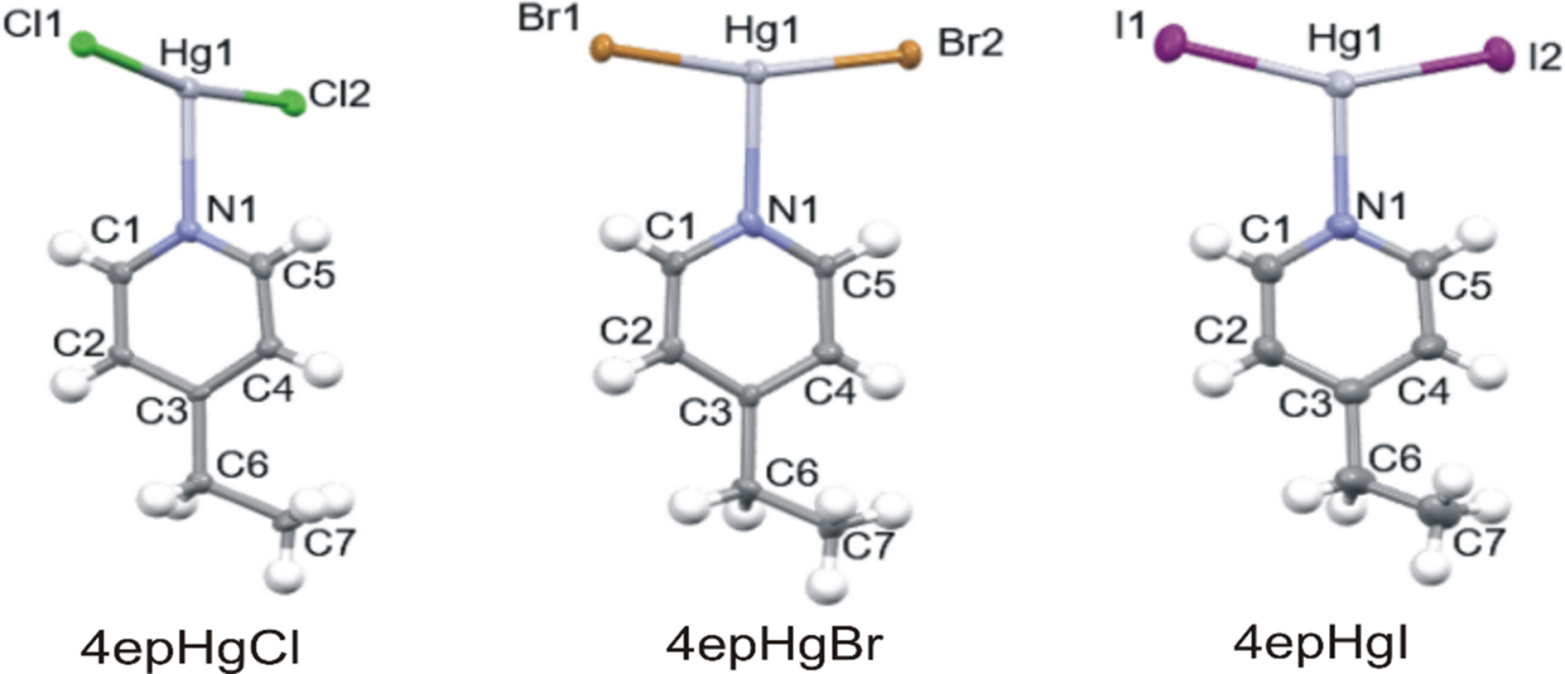
The asymmetric units of **4epHgCl**, **4epHgBr** and **4epHgI**, showing the atomic numbering schemes. Displacement ellipsoids are drawn at the 50% probability level for all the structures and H atoms are shown as small spheres of arbitrary radii.

Three new crystal structures containing 4-ethyl­pyridine (4-Etpy) with Hg^II^ halides as the inorganic portions were determined, all displaying one-dimensional halide-bibridged polymeric structures, in which the Hg^II^ cations are bridged by two halide ligands. The crystallographic parameters of these structures are listed in Table 1 ▸ and their asymmetric units are illustrated in Fig. 1 ▸. Selected bond lengths and bond angles are given in Table S1 and weak C—H⋯*X* hy­dro­gen-bonding inter­actions in Table S2 in the supporting information.

### Crystal structures of 4epHgCl, 4epHgBr and 4epHgI

Similar one-dimensional halide-bridged polymers were ob­tained for **4epHgCl**, **4epHgBr** and **4epHgI**; however, they are not isostructural. **4epHgCl**, **4epHgBr** and **4epHgI** crystallize in the space groups *P*2_1_2_1_2_1_*, P*2_1_/*c* and *Fdd*2, respectively. The asymmetric units of all three structures consist of a Hg^II^ ion coordinated to two halide ligands and one 4-ethyl­pyridine organic ligand coordinated *via* the N atom, as illustrated in Fig. 1 ▸.

**Figure 2:**
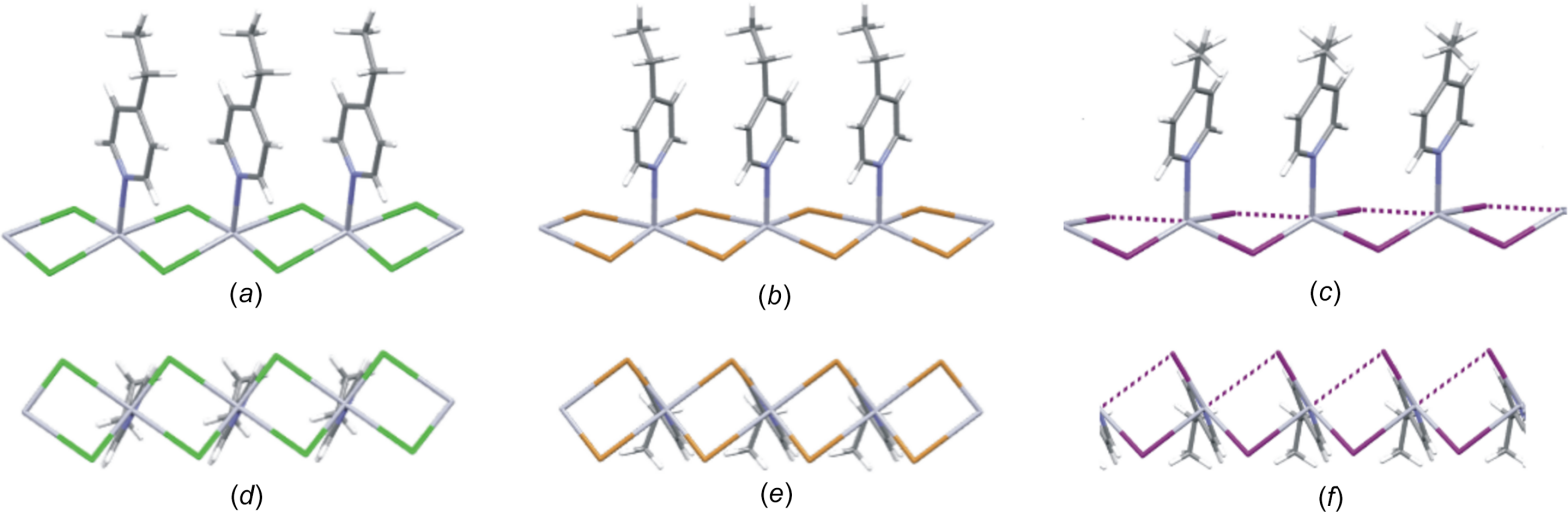
The halide-bibridged polymer chains in (*a*) **4epHgCl**, (*b*) **4epHgBr** and (*c*) **4epHgI**. The pseudo-parallelogram inorganic portion and the organic ligand tilt in (*d*) **4epHgCl** and (*e*) **4epHgBr**, and (*f*) the inorganic portion and organic ligand tilt in **4epHgI**. The dotted lines in parts (*c*) and (*f*) indicate longer and weaker Hg⋯I bonding interaction in the polymeric structure.

Repetition of the asymmetric unit results in the formation of a one-dimensional halide-bibridged polymer in all three structures, in which pairs of Hg^II^ ions are bridged by two halide ligands, as illustrated in Figs. 2 ▸(*a*)–(*c*). The polymer adopts a scalloped ribbon conformation, with all the organic ligands coordinated to the same side of the polymer chain, as shown in Figs. 2 ▸(*d*)–(*f*). In these polymers, the Hg^II^ ion adopts a square-pyramidal geometry, with four equatorial halide ligands and the 4-ethyl­pyridine ligand as the axial ligand. It was found that the basal plane of the square pyramid is not coplanar with the Hg^II^ ion in all three structures, indicating a slight distortion in the geometry.

In all the structures, the metal halide portion displays a puckered pseudo-parallelogram geometry, consisting of four unequal Hg—*X* bonds, in which the two opposite Hg—*X* bond lengths are similar, with two Hg—*X* bonds being shorter and two longer. Shorter Hg—*X* bonds alternate with longer Hg—*X* bonds, with similar shorter Hg—*X* bond lengths of 2.3471 (18) and 2.3562 (18) Å in **4epHgCl**, 2.4732 (8) and 2.4815 (8) Å in **4epHgBr**, and 2.6269 (6) and 2.6576 (6) Å in **4epHgI**. The polymer chain in **4epHgI** adopts a similar pattern to that of **4epHgCl and 4epHgBr**, but does not consist of the repeating pseudo-parallelogram motif, but rather features open pseudo-parallelogram units, as illustrated in Fig. 2 ▸(*f*). The longer Hg—*X* bonds may be viewed as semi-coordinated inter­actions, and have values of 3.086 (2) and 3.094 (2) Å in the chloride structure, 3.1704 (9) and 3.2694 (2) Å in the bromide structure, and 3.1949 (6) and 3.8988 (8) Å in the iodide structure. The presence of the very long semi-coordinated Hg—I bond of 3.8988 (8) Å in **4epHgI** causes the iodide-bibridged polymer to have a dis­torted geometry com­pared to the polymers in **4epHgCl** and **4epHgBr**, as can be seen in Figs. 2 ▸(*d*)–(*f*) (Slabbert *et al.*, 2015*b* ▸). The puckered geometry of the halide-bridged polymers in related com­pounds has been reported previously and is a mechanism to shorten the Hg⋯Hg distance, and thus the distance between the aromatic planes of the organic ligands, to allow for the formation of aromatic inter­actions between the organic ligands (Slabbert *et al.*, 2015*b* ▸).

As the size of the halide ligand increases from chloride to bromide to iodide, the Hg—*X* bond lengths increase, resulting in an increase in the Hg⋯Hg distance in the halide-bibridged polymer. The Hg⋯Hg distance is 3.9382 (6) Å in **4epHgCl**, 4.0679 (6) Å in **4epHgBr** and 4.3247 (5) Å in **4epHgI**, indicating very weak mercurophilic inter­actions (Kumar *et al.*, 2013 ▸; Doerrer *et al.*, 2010 ▸) in the polymer chain. The Hg—N bond lengths decrease with an increase in halide ligand size, with Hg—N bond lengths of 2.441 (6) Å in **4epHgCl**, 2.436 (7) Å in **4epHgBr** and 2.373 (7) Å in **4epHgI**.

Along the halide-bibridged polymer, the two opposite angles *X*1—Hg—*X*1 and *X*2—Hg—*X*2 have larger values than the *X*1—Hg—*X*2 angles within the polymer, with values of 91.51 (6) and 91.88 (6)° for the larger angles, and 87.23 (6) and 87.25 (6)° for the smaller angles in **4epHgCl**. In the bromide analogue, **4epHgBr**, the two opposite angles *X*1—Hg—*X*1 and *X*2—Hg—*X*2 are 91.23 (2) and 89.09 (2)°, respectively, while the angles inside the polymer, *X*1—Hg—*X*2, are 89.34 (2) and 86.96 (2)°. The *X*2—Hg—*X*2 angle is 94.841 (18)° and the *X*1—Hg—*X*2 angle is 96.216 (19)° in **4epHgI**, with a semi-coordinated Hg⋯I inter­action of 3.8988 (8) Å. The Hg—*X*—Hg angles are 91.51 (6) and 91.88 (6)° in **4epHgCl**, 91.23 (2) and 89.09 (2)° in **4epHgBr**, and 94.841 (18)° in **4epHgI.** As the size of the halide ligand increases, the inter-strand *X*⋯*X* distances also increase from 3.792 (3) Å in **4epHgCl** to 3.998 (1) Å in **4epHgBr** to 4.3503 (9) Å in **4epHgI**. This difference might be the reason for the formation of a less-sym­metric structure as the size of the bridging halide increases (Hu *et al.*, 2007 ▸).

While the aromatic groups of the ligands are coplanar in all the structures, the one-dimensional polymers differ in terms of the relative orientation of their methyl substituents, as illustrated in Figs. Figs. 2 ▸(*d*)–(*f*). This difference in orientation is also evidenced by the C2—C3—C6—C7 and C4—C3—C6—C7 torsion angles of 170.2 (7) and −12.1 (12)° in **4epHgCl**, −130.2 (9) and 50.0 (12)° in **4epHgBr** and −147.0 (12) and 32.1 (19)° in **4epHgI**. Furthermore, the perpendicular distance between the plane containing the aromatic ring and the terminal aliphatic C7 atom increases from 0.262 Å in **4epHgCl** to 1.078 Å in **4epHgBr**; however, this distance decreases to 0.766 Å in **4epHgI**, indicating that the spatial orientation of the ethyl group of the organic ligand is influenced by the size of the halide ligand in the polymeric chain.

**Figure 3:**
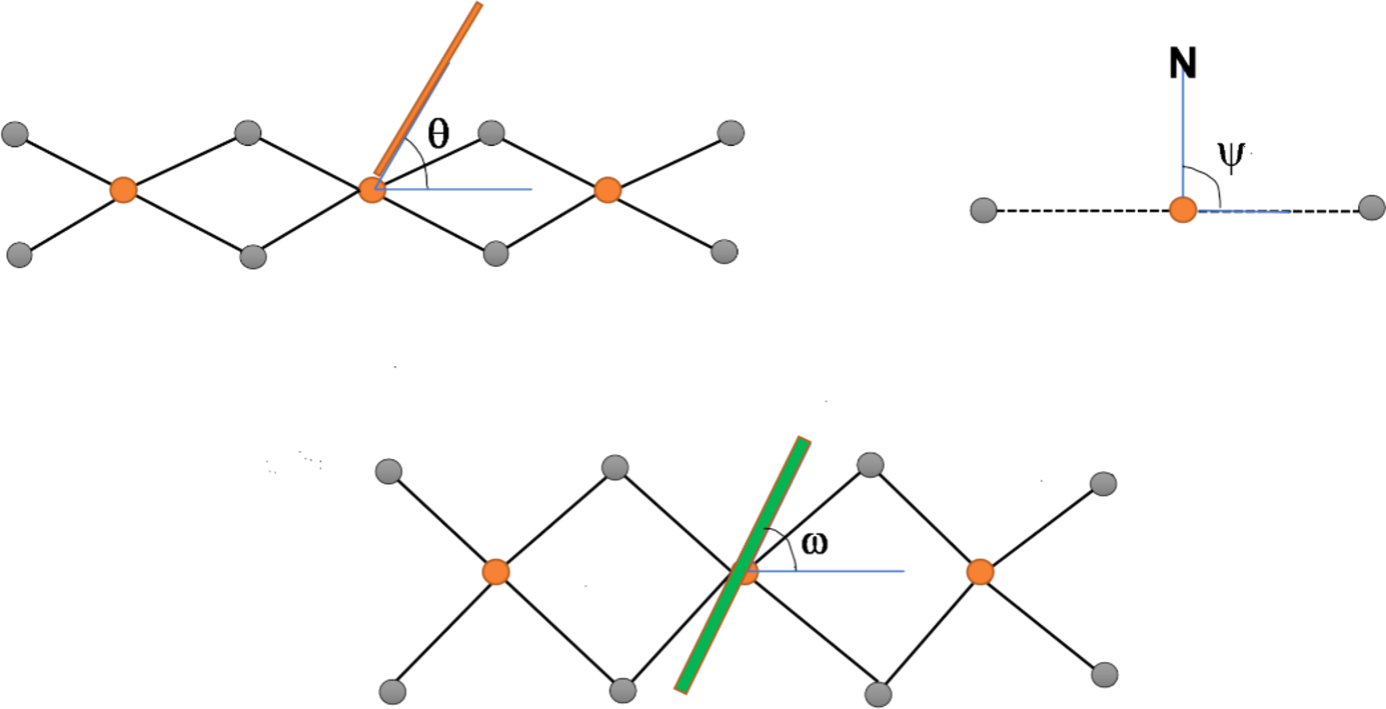
The descriptor angles θ, ψ and ω adapted from Hu *et al.* (2003 ▸). The orange spheres represent the metal cations, the grey spheres represent the bridging halide ligands and the thick orange and green lines represent the plane that contains the aromatic ring.

In order to com­pare the geometric features of the one-dimensional halide-bibridged polymers for systems with com­position [Hg(μ-*X*)_2_(*L*)_2_]_*n*_, the structural descriptor angles θ, ψ and ω, originally defined by Hu *et al.* (2003 ▸), are used in this study, with small modifications made to the descriptors, as explained below. The descriptors are shown in Fig. 3 ▸. The angle θ indicates the orientation of the organic N-donor ligand relative to the one-dimensional halide-bridged chain, while the angle ψ is defined as the angle between the N atom, the metal ion to which it is coordinated and the adjacent metal centre (N—*M*1⋯*M*2). The angle, ω, between the aromatic ring plane and the metal centre plane that passes through the Hg metal centres of equivalent halide-bridged polymers gives an indication of the degree of tilt of the aromatic group of the organic ligand relative to the halide-bridged polymer, as shown in Figs. 2 ▸(*d*) and 2(*e*). The schematic representation of the relative orientation of the aromatic ligand with respect to the halide-bridged polymer, represented by θ, ψ and ω, are shown in Fig. 3 ▸.

The angle between the aromatic plane of the organic ligand and the plane through the halide ligands, θ, is 80.96° in **4epHgCl**, 89.40° in **4epHgBr** and 75.74° in **4epHgI**. The structures of **4epHgCl**, **4epHgBr** and **4epHgI** exhibit ω angles of 65.31, 60.01 and 66.67°, respectively. There is a slight decrease in the ω angle from **4epHgCl** to **4epHgBr**, indicating that there is a larger degree of organic ligand rotation with the increase in size of the halide ligand. When the halide ligand changes to iodide, this trend does not continue, since a different structure is formed, as shown in Fig. 2 ▸(*f*), indicating that the size of the halide ligand in the halide-bridged polymer affects the con­formation and rigidity of the polymer chain and this influences the orientation of the organic ligand attached to the chain. As the size of the halide ligand increases from chloride to iodide, the angle between the two metal-centre planes, containing the Hg^II^ cation, *X*1 and *X*2 in adjacent pseudo-parallelogram units, increases significantly from 15.60° in **4epHgCl** to 19.82° in **4epHgBr** to 30.50° in **4epHgI**, indicating increasing spatial distortion and flexibility of the Hg—*X*—Hg bridge.

The centroid-to-centroid distance between the pyridine moieties of the organic ligands is 3.938 Å in **4epHgCl**, 4.068 Å in **4epHgBr** and 4.325 Å in **4epHgI**, indicating weak aromatic interactions between neighbouring pyridine rings (Janiak *et al.*, 2000 ▸). It should be noted that the centroid-to-centroid distance depends on the halide-bridge lengths. As the size of the halide ligand increases from chloride to bromide to iodide, both the Hg—*X* and the Hg⋯Hg distances increase, causing an increase in the cen­troid-to-centroid distances between the pyridine moieties of the organic ligands. The ψ angle is 85.9 (1)° for **4epHgCl**, 88.0 (2)° for **4epHgBr** and 83.1 (2)° for **4epHgI**. The observed values of the ψ and θ angles in these halide-bibridged polymers indicate that the organic ligands are not perpendicular to the inorganic plane. This orientation has been adopted to ensure stability of the polymer *via* weak C—H⋯*X* hy­dro­gen-bonding inter­actions, as listed in Table S2 in the supporting information. As can be seen from Table S2, both the donor–acceptor (*D*⋯*A*) distances and *D*—H⋯*A* angles increase as the size of the halide ligand increases. This results in weaker hy­dro­gen-bonding inter­actions as the size of the halide increases and hence looser mol­ecular packing of the polymeric chains within the crystal lattice.

**Figure 4:**
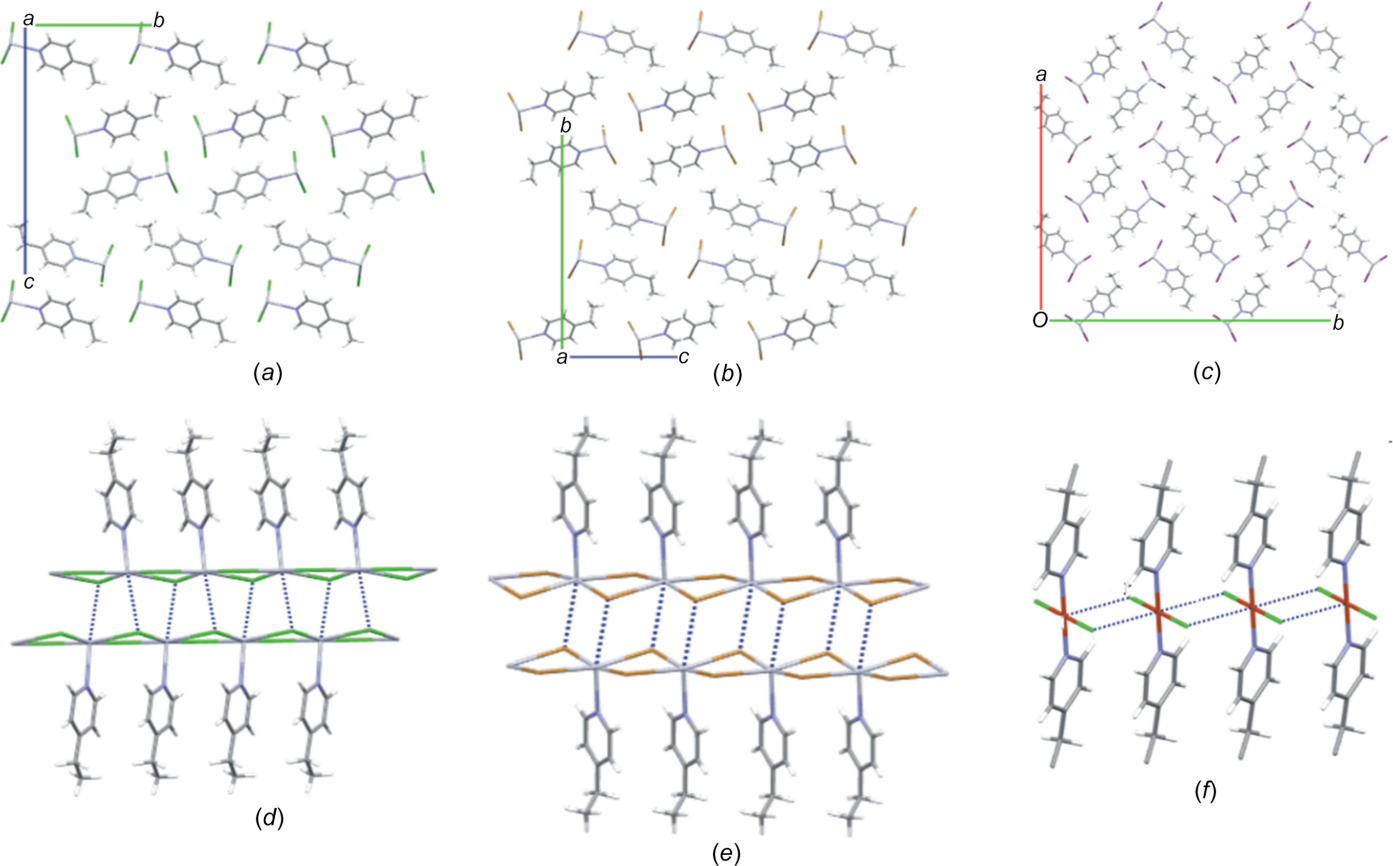
Packing diagrams of (*a*) **4epHgCl**, (*b*) **4epHgBr** and (*c*) **4epHgI**. The long semi-coordinated inter­actions forming the pseudo-octa­hedral polymer in (*d*) **4epHgCl** and (*e*) **4epHgBr**. (*f*) The halide-bibridged polymer in **4epCuBr** (CSD refcode CEPYCU; Laing *et al.*, 1971 ▸).

The packing diagrams are illustrated in Figs. 4 ▸(*a*)–(*c*). Pairs of the halide-bridged polymers pack in a head-to-tail fashion, forming a layered structure in **4epHgCl** and **4epHgBr**, and a checkerboard pattern in **4epHgI**. The one-dimensional halide-bibridged polymers in the structures of **4epHgCl** and **4epHgBr** pack in such a way that the halide-bridged portions of two neighbouring polymeric chains approach each other, as shown in Figs. 4 ▸(*d*) and 4(*e*). This allows for the formation of long semi-coordinated Hg⋯*X*⋯Hg inter­actions to neighbouring polymer chains, as illustrated in Figs. 4 ▸(*d*) and 4(*e*), resulting in a pseudo-one-dimensional halide-bridged polymer that shows octa­hedral coordination of the Hg^II^ ions, which com­pletes the coordination of the Hg^II^ ion. The Hg⋯*X* contact dis­tances are 3.394 (2) Å in **4epHgCl** and 3.5909 (9) Å in **4epHgBr**, indicating that the Hg⋯*X* inter­actions between neighbouring polymer chains become weaker as the size of the halide ligand increases from chloride to bromide. This type of inter­chain inter­action between polymers of this type is also seen in the structure com­prised of HgBr_2_ and phenazine organic ligands (Slabbert *et al.*, 2015*b* ▸). The packing arrangement in **4epHgI** is different to that of **4epHgCl** and **4epHgBr**, with adjacent polymer chain pairs rotated 90° relative to each other and with no inter­chain Hg⋯I inter­actions between neighbouring polymer chains. This means that the change of the halide ligand from chloride to bromide, with the organic ligand and metal ion remaining constant, can be accommodated in the specific structural type; however, the change to the larger iodide ligand represents a tipping point that requires a change to a different structure type.

The CuBr_2_ analogue of **4epCuBr** has been reported (CSD refcode CEPYCU; Laing *et al.*, 1971 ▸); however, this com­pound exhibits a halide-bibridged polymer with a flat metal–halide portion, in which the Cu^II^ ion displays a tetra­gonal geometry due to Jahn–Teller distortion, with the 4-Etpy organic ligand coordinating to both sides of the halide-bibridged polymer, as illustrated in Fig. 4 ▸(*f*).

## Conclusions

**4epHgCl**, **4epHgBr** and **4epHgI** display similar one-dimensional scalloped halide-bridged polymeric structures involving two halide ligands bridging two metal centres, in which the Hg^II^ ion adopts a coordination number of five. The size of the halide ligand has a significant effect on the structure, geometry and packing arrangement of the polymer chains, with **4epHgI** displaying a different packing motif com­pared to the chloride and bromide analogues. In the structures of **4epHgCl** and **4epHgBr**, the polymer chains further associate *via* long semi-coordinated Hg⋯*X* inter­actions to form a pseudo-octa­hedral polymer. As the size of the halide ligands increases, the Hg—*X* bond length within the polymer chain increases, causing structural distortion in the polymer chain. The change of the halide ligand to the larger iodide ligand disrupts the formation of the regular halide-bibridged polymeric chain observed in the chloride and bromide analogues, with **4epHgI** displaying pseudo-bridging in the polymer chain.

## Supplementary Material

Crystal structure: contains datablock(s) 4epHgCl_updated, 4epHgBr_updated, 4epHgI_updated, global. DOI: 10.1107/S2053229625009702/eq3024sup1.cif

Structure factors: contains datablock(s) 4epHgCl_updated. DOI: 10.1107/S2053229625009702/eq30244epHgCl_updatedsup2.hkl

Structure factors: contains datablock(s) 4epHgBr_updated. DOI: 10.1107/S2053229625009702/eq30244epHgBr_updatedsup3.hkl

Structure factors: contains datablock(s) 4epHgI_updated. DOI: 10.1107/S2053229625009702/eq30244epHgI_updatedsup4.hkl

Selected geometry. DOI: 10.1107/S2053229625009702/eq3024sup5.pdf

CCDC references: 2442692, 2442693, 2442694

## Figures and Tables

**Table 1:** Experimental details Experiments were carried out at 150 K with Mo *K*α radiation using a Bruker PHOTON 100 CMOS diffractometer. Absorption was corrected for by multi-scan methods (*SADABS*; Bruker, 2013 ▸). H-atom parameters were constrained.

	**4epHgCl**	**4epHgBr**	**4epHgI**
Crystal data			
Chemical formula	[HgCl_2_(C_7_H_9_N)]	[HgBr_2_(C_7_H_9_N)]	[HgI_2_(C_7_H_9_N)]
*M*_r_	378.64	467.54	561.54
Crystal system, space group	Orthorhombic, *P*2_1_2_1_2_1_	Monoclinic, *P*2_1_/*c*	Orthorhombic, *Fdd*2
*a*, *b*, *c* (Å)	3.9382 (5), 10.3571 (15), 22.758 (3)	4.0679 (5), 22.583 (3), 10.9050 (14)	30.3836 (14), 34.6159 (16), 4.3247 (2)
α, β, γ (°)	90, 90, 90	90, 95.488 (4), 90	90, 90, 90
*V* (Å^3^)	928.3 (2)	997.2 (2)	4548.5 (4)
*Z*	4	4	16
μ (mm^−1^)	17.09	23.39	18.91
Crystal size (mm)	0.51 × 0.23 × 0.12	0.42 × 0.18 × 0.10	0.23 × 0.06 × 0.06

Data collection			
*T*_min_, *T*_max_	0.068, 0.260	0.244, 0.745	0.366, 0.747
No. of measured, independent and observed [*I* > 2σ(*I*)] reflections	16644, 1876, 1869	21065, 2024, 1908	30677, 2271, 2251
*R*_int_	0.058	0.062	0.038
(sin θ/λ)_max_ (Å^−1^)	0.625	0.625	0.625

Refinement			
*R*[*F*^2^ > 2σ(*F*^2^)], *wR*(*F*^2^), *S*	0.020, 0.053, 1.11	0.033, 0.094, 1.08	0.018, 0.045, 1.15
No. of reflections	1876	2024	2271
No. of parameters	103	96	102
No. of restraints	0	0	1
H-atom treatment	H-atom parameters constrained	H-atom parameters constrained	H-atom parameters constrained
Δρ_max_, Δρ_min_ (e Å^−3^)	1.29, −1.22	2.19, −1.85	1.17, −0.46
Absolute structure	Refined as an inversion twin	–	Refined as an inversion twin
Absolute structure parameter	0.186 (14)	–	0.026 (7)

Computer programs: *APEX2* (Bruker, 2013 ▸), *SAINT* (Bruker, 2013 ▸), *SHELXT2013* (Sheldrick, 2015*a* ▸) and *SHELXL2013* (Sheldrick, 2015*b* ▸) in *WinGX* (Farrugia, 2012 ▸), *Mercury* (Macrae *et al.*, 2020 ▸), *PLATON* (Spek, 2020 ▸) and *OLEX2* (Dolomanov *et al.*, 2009 ▸).
