# Supplementary material for: Effect of the size of halide ligands on the crystal structures of halide-bibridged polymers of HgX2 with 4-ethyl­pyridine
Source: Acta Crystallogr C Struct Chem. 2025 Nov 10;81(Pt 12):680–6. doi: 10.1107/S2053229625009702 (PMC12805918; doi:10.1107/S2053229625009702)
Supplement: Supplementary file 5 [file c-81-00680-sup5.pdf]

STRUCTURAL CHEMISTRY

Volume 81 (2025)

Supporting information for article:

Effect of the size of the halide ligands on the crystal structures of halide-bibridged polymers of Hg*X*2 with 4-ethylpyridine

B. M. Parveen Beebeejaun-Boodoo

Supporting information

Table S1 Selected bond lengths, angles and torsion angles in structures **4epHgCl, 4epHgBr, 4epHgI** (Å, °).

	**4epHgCl**	**4epHgBr**	**4epHgI**
	M=Hg, X=Cl	M=Hg, X=Br	M=Hg, X=I
M1−N1 (Å)	2.441(6)	2.436(7)	2.373(7)
M1−X1 (Å)	2.3562(18)	2.4815(8)	2.6269(6)
M1−X2 (Å)	2.3471(18)	2.4732(8)	2.6576(6)
M1−X2^{i}(Å)	-	-	3.1949(6)
X2−M1^{ii}(Å)	-	-	3.1949(6)
N1−M1−X2(°)	98.16(15)	98.28(16)	101.05(17)
N1−M1−X1 (°)	94.51(15)	97.73(16)	105.63(17)
X2−M1−X1 (°)	-	163.92(3)	151.07(19)
X2^{i}−M1−X1^{i} (°)			96.216(19)
N1−M1−X2^{i}	-	-	89.85(18)
X1−M1−X2^{i}	-	-	96.841(18)
X2−M1−X2^{i}	-	-	94.841(18)
M1−X2−M1^{ii}	-	-	94.844(15)
C5−N1−M1(°)	120.6(5)	121.2(5)	119.8(6)
C1−N1−M1(°)	120.8(5)	121.1(6)	122.2(5)
C7−C6−C3 (°)	115.9(7)	114.0(7)	116.5(8)
M1−N1−C1−C2 (°)	175.2(5)	-178.2(6)	-175.3(8)
M1−N1−C5−C4 (°)	-174.1(6)	176.2(6)	174.8(9)

i: ¼ -x, 1/4+y, 1/4+z ii: x+1/4,-y+1/4,z-3/4

Table S2 Weak C-H···X hydrogen bonding parameters [°] for structures **4epHgCl**, **4epHgBr** and **4epHgI**.

**Structure**	**D-H···A (Å)**	**d(D−H) (Å)**	**d(H···A) (Å)**	**d(D···A) (Å)**	**∠ (DHA) (°)**	**Symmetry Operator**
**4epHgCl**	C1−H1···Cl1	0.95	2.88	3.527(8)	126.2	
**4epHgBr**	C1−H1···Br1	0.95	3.04	3.708(9)	128.7	i: -x+2,-y+1,-z+1
	C2−H2···Br1^{i}	0.95	3.03	3.961(8)	165.5	
	C5−H5···Br2	0.95	3.04	3.723(8)	129.6	
**4epHgI**	C5−H5···I2	0.95	3.21	3.883(9)	129.8	ii: x+1/4,-y+1/4,z-3/4
	C5−H5···I1^{ii}	0.95	3.32	4.130(9)	144.1	
